# Impact of peak estradiol levels on reproductive outcome of intracytoplasmic sperm injection

**DOI:** 10.12669/pjms.305.5175

**Published:** 2014

**Authors:** Rehana Rehman, Shireen Jawaid, Hina Gul, Rakhshaan Khan

**Affiliations:** 1 Dr. Rehana Rehman, PhD, Assistant Professor, Department of Biological & Biomedical Sciences, Aga Khan University, Karachi – Pakistan.; 2 Dr. Shireen Jawed, M.Phil, Assistant Professor of Physiology, Islam Medical & Dental College, Sialkot, Pakistan.; 3 Hina Gul, Senior Research Analyst, SMAR International (Pvt.) Ltd,; 4 Dr. Rakhshaan Khan, Public Health Physician,

**Keywords:** Peak Estradiol, Controlled ovarian stimulation, Intracytoplasmic sperm injection

## Abstract

***Objective: ***To observe effect of peak estradiol (E2) levels on outcome after intra cytoplasmic sperm injection (ICSI).

***Methods: ***Quasi experimental design conducted in “Islamabad Clinic Serving Infertile Couples” from June 2010 till August 2011. Down regulation with mid luteal suppression of 564 females aged 18-41 years was done with gonadotrophin releasing hormone agonist followed by controlled ovarian stimulation, ovulation induction (OI), oocytes pickup and embryo transfer. Samples for peak serum E2 at the time of OI was estimated by Enzyme Linked Immuno Sorbent Assay. The association of peak E2 with outcome groups of Intracytoplasmic sperm injection (ICSI) (Group I) beta hCG <5 m IU/ml, (Group II) with beta hCG>5 mI U/ml and no cardiac activity and (Group III) with beta hCG>5mIU/ml and cardiac activity on trans vaginal scan was identified. Statistical comparison by one way analysis of variance (ANOVA) via SPSS version 15 was done.

***Results: ***A clinical pregnancy rate of 36% was achieved, pregnant females(Group III) had higher peak E2 2269 ± 80.97 as compared to 1419 ± 37.07 and 1807±90.28 pg/ml (mean ± SD) in Groups I and II respectively (p<0.0001) Elevated serum E2 was significantly associated with better oocyte recovery, fertilization, cleavage and implantation rates (p<0.0001) .

***Conclusion: ***A high serum E2 measured on OI day can predict success of treatment after ICSI. Females who had high peak E2 had greater number of retrieved, mature and fertilized oocytes with thick endometrial lining that helped in implantation and clinical pregnancy.

## INTRODUCTION

Infertility is becoming a health challenge worldwide. It has been documented that infertility rate in Pakistan is 21.9% with 3.5% cases of primary and 18.4% cases of secondary infertility.^1^ This is catered by assisted methods of reproduction; In vitro fertilization (IVF) and Intracytoplasmic Sperm Injection (ICSI). The latter has emerged as micro-manipulation technique, in which single sperm is injected into a single egg in order to achieve fertilization.^2^

Multiple factors have been suggested for prediction of better outcomes in ICSI cycles such as agonist/antagonist down regulation, selection of drug used for controlled ovarian stimulation (COS), age, antral follicle count, basal FSH, number of injected oocytes, fertilization rate, embryo morphology, peak and mid luteal estradiol (E2), progesterone (P) levels and quality of transferred embryos.^3^

During the reproductive years estradiol (E2) in women is produced mainly by the granulosa cells of the ovaries through the aromatization of androstenedione to estrone. It is necessary for endometrial preparation, blastocysts implantation and progesterone priming of endometrium.^4^ Peak E2 levels measured on the day of hCG administration thus remains an integral component of assessing response to COS.^5^ Conflicting results regarding the effect of peak E2 on outcome of intracytoplasmic insemination have been reported by different researchers. It was considered to be a poor predictor of IVF outcome.^6^ Some studies do not support an association between peak E2 levels and pregnancy outcome,^7^^-^^10^ whereas other studies suggested higher peak E2 levels with better pregnancy rates achieved.^11^^,^^12^ Objective of this study was to evaluate the effect of peak E2 on pregnancy and implantation rates in patients undergoing ICSI in an infertility clinic.

## METHODS

Quasi experimental design conducted after approval from Ethical Review “Board of Karachi University” and “Islamabad Clinic Serving Infertile Couples” from June 2010 till August 2011. Patients were recruited by convenience sampling during the study period after which a written informed consent was acquired by the couple. Females were included on the basis of; age 20-41 years, duration of infertility more than 3 years, both ovaries present with no morphological abnormalities, normal ovulatory cycle (25–35 days), body mass index (BMI)of 18–35 kg/m^2^, basal FSH (day 2) serum level < 9I U/ mL, long protocol treatment with Gonadotrophin releasing hormone agonist (Gn Rh), stimulated with injection of recombinant follicle stimulating hormone (r FSH; Puregon) and prescribed vaginal progesterone support with 400 mg pessaries (cyclogest). Females with polycystic ovaries, fibroids, on Gn Rh antagonist, short down regulation with GnRH agonist and ICSI with sperm retrieval by testicular biopsy were excluded.

**Fig.1:**
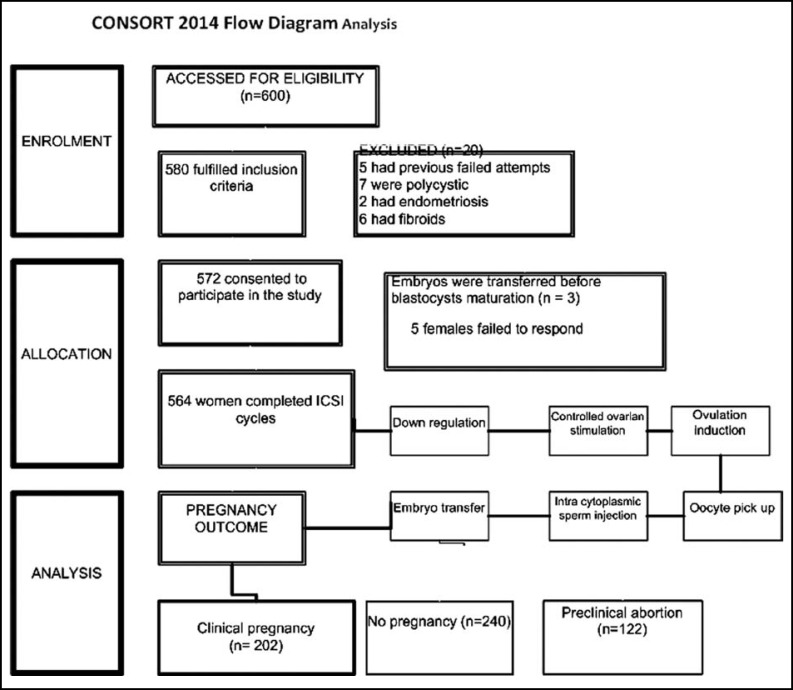
Flow chart representing treatment protocol

***Treatment Protocol: ***Females were down regulated with daily injection Deca Peptyl (Gonadotrophin releasing hormone agonist) from Day 21 of previous cycle followed by COS with gonadotrophin (Injection Puregon S/C) from 2^nd^ to third day of cycle for fourteen days. Maturity of follicle was assessed by series of Trans vaginal scan (TVS) started from 5^th^ day of COS till decision of oocyte pick up (OPU). Ovulation induction (OI) with intra muscular injection of hCG (Pregnyl 10,000 I.U) was performed COS 12 ± 1 days with majority of follicle acquiring a size of 20mm. Samples for peak. E2 taken by venipuncture on the day of OI were analyzed by ELISA radioimmunology assay. Oocytes were retrieved 36 hours after OI by vaginal ultrasound probe with 16G adapter and double lumen oocyte aspiration needle. Oocytes were, treated and then transferred to the incubator for about 1-2 hours prior to insemination by ICSI procedures. Semen analysis was performed by strict Kruger’s criteria.^13^^,^^14^ Micro injections of spermatozoa were performed at right angles to the position of polar body under the microscope. Fertilization, (presence of two pronuclei; 2PN), grading cleavage till differentiation of embryo into blastocyst was checked. Embryo transfer (ET) of blastocysts was done seven days after OI by Sims-Wallace Embryo Replacement Catheter under ultrasound guidance (Fig.1). Luteal support was maintained by progesterone vaginal pessaries (Cyclogest 400 mg) twice a day from the day of OPU.

***Outcome measures: ***Serum beta hCG measurement and TVS performed14 and 28 days after OPU (respectively)categorized females into groups; (I), non pregnant with beta hCG<5 mI U/ml, (II); preclinical abortion beta hCG>5 mIU/ml with no fetal cardiac activity on TVS, (III); clinical pregnancy with beta hCG>5 mIU/ml and cardiac activity confirmed by TVS.

Pregnancy outcomes and the associated rates (in percentages) were defined using standard definitions as follows. Oocyte recovery rate was of number of oocytes retrieved in comparison to number of follicles calculated.^5^ Fertilization rate (FR) was proportion of micro injected oocytes resulting in two pronuclei formation.^15^ The implantation rate (IR) was the number of gestational sacs visualized on TVS divided by the number of embryos transferred.^4^ A pregnancy rate (PR) was calculated by presence of an intrauterine gestational sac observed on TVS per number of patients in the cycle.^5^

***Statistical Analysis: ***Data analysis was done via SPSS (Statistical Packages of Social Sciences) version 15.0. Clinical characteristics were summarized in terms of frequencies and percentages for qualitative variables (age group), meanSD for continuous/quantitative variables. Statistical comparison of peakE2 in all outcome groups was performed by using one way analysis of variance (ANOVA). In all statistical analysis only p-value <0.05 was significant. The different rates of reproductive outcome were compared with peak E2 by linear and logistic regression analysis.

## RESULTS

**Fig.2:**
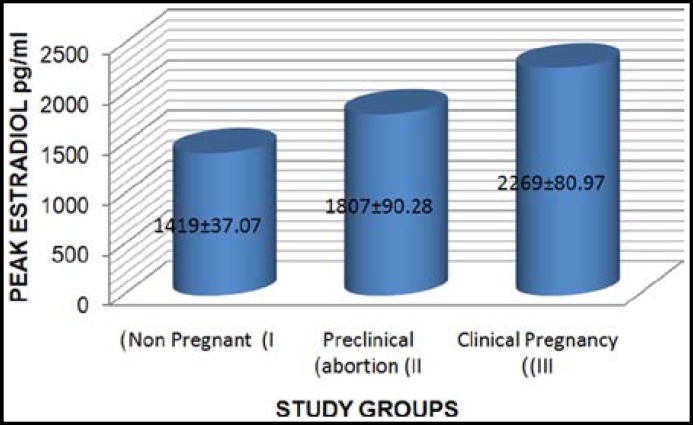
Comparison of Peak E2 in study groups

Results of 564 patients represent female age 31.55± 4.62 years, duration of infertility 7.48 ±.3.68 years with 80% cases of primary and 20% suffering from secondary infertility). Group I had 240 (42%) non pregnant females, 122 females (22%) in Group II had preclinical abortion whereas results of Group III showed 202 females with clinical pregnancy (36% PR). Comparison of serum peak E2 levels (pg/ml) in Group I (1419±37.07), II (1807±90.28) and III (2269±80.97) was found to be significantly high in Group III (p value 0.000) (Fig.2). The comparison of oocyte parameters, endometrial thickness and reproductive rates of all the groups given in (Table-I) represents oocyte quality and reproductive outcome in females of all groups.

Comparison of peak E2 with multiple regression analysis showed significant positive association of peak E2with implantation rate (Table-II). Logistic linear regression of peak E2 with clinical pregnancy rate showed E2 (OR=1.00; SE=0.00, P=0.000).

## DISCUSSION

The peak E2 in natural menstrual cycles typically decrease after the initial LH surge with feedback inhibition of hypothalamo- pituitary -ovarian axis, optimal mono follicular development and oocyte maturation^16^ In COS, normal axis is disrupted to produce multiple follicles and oocytes at different stages of maturation for better reproductive outcome.^6^^,^^17^ The determination of peak E2 value can thus be important to estimate the success of ICSI procedure with respect to follicular development, maturation and endometrial receptivity.

**Table-I:** Comparison of oocyte &embryological details

	***Group I*** ***Not pregnant*** ***n=240***	***Group II Preclinical abortion*** ***n= 122***	***Group III Clinical pregnancies*** ***n=202***
Preovulatory follicle count	17.992 ± 8.88	18.262 ± 8.606	22.792 ±7.785^** °°,^
No of oocytes/patient	17.708 ± 8.969	17.852 ± 8.522	22.218 ± 7.84^** °°,^
No of oocytes Metaphase II	12.5 ±7.548	14.164 ± 8.234	20 ± 7.693^** °°,^
No of oocytes fertilized	12.1 ±7.407	13.918 ±8.385	19.307 ± 7.519^** °°,^
No of cleaved embryos	6.4 ±4.318	9.869±6.637	15.941±6.367^** °°,^
No of transferred embryos	1.65 ±0.575	1.459 + 0.594	1.703 + 0.575*
Endometrial thickness	5.967 ± 2.691	9.033 ±2.345	11.455 ±2.037^** °°,^
Number of gestational sacs	0	0.052 ±0.223	1.248 ± 0.639^** °°,^
Oocyte retrieval rate (% age)	97.52 ±7.088	97.162 ±8.011	96.882 ±5.769
Oocyte maturity rate(% age)	70.853 ± 22.305	78.575 ± 17.829	89.676 ± 10.742^** °°,^
Fertilization rate (% age)	68.886 ± 23.202	76.504 ±18.919	86.698±12.207^** °°,^
Cleavage rate (% age)	36.573 ±16.163	54.29 ±23.272	71.876 ±12.638^** °°,^
Implantation. rate (% age)	0	4.918 ±21.804	75.248 ±33.88^** °°,^

Values expressed as Mean ± SD,

Groups compared by one way analysis of variance,

*, p value <0.01, for significant results of Clinical pregnancy with Preclinical abortion

**, p value < 0.001 for significant results of Clinical pregnancy with Preclinical abortion

°°, p value < 0.001 for significant results of Clinical pregnancy with not pregnant group

Oocyte retrieval rate (%)= Total number of follicles at ultrasound/number of oocytes retrieved × 100

Oocyte maturity rate(%)= Total number of metaphase II oocytes / Total number of oocytes retrieved × 100

Fertilization rate (%)= Total number of 2 pronuclei/ Total number of oocytes microinjected × 100

Cleavage rate(%)= Total number of embryos cleaved / Total number of 2 pronuclei× 100

Implantation rate (%)= Total number of gestational sacs/ Total number of embryos transferred × 100

**Table-II:** Influence of peak Estradiol on reproductive rates of patients undergoing Intracytoplasmic Sperm injection

***Reproductive Rates***	***B***	***Std. Error***	***R Square***	***Sig.***
Oocyte retrieval rate (%)	0.000	0.000	0.004	.284
Oocyte maturity rate (%)	-0.0003	0.0008	0.0004	.731
Fertilization rate (%)	0.0000	0.001	0.000	.990
Cleavage rate (%)	0.0016	0.0009	0.0122	.064
Implantation rate (%)	0.0048	0.0016	0.0313	.003

Linear regression analysis:

Oocyte retrieval rate (%)= Total number of follicles at ultrasound/number of oocytes retrieved × 100

Oocyte maturity rate(%)= Total number of metaphase II oocytes / Total number of oocytes retrieved × 100

Fertilization rate (%)= Total number of 2 pronuclei/ Total number of oocytes microinjected × 100

Cleavage rate(%)= Total number of embryos cleaved / Total number of 2 pronuclei× 100

Implantation rate (%)= Total number of gestational sacs/ Total number of embryos transferred × 100

Mean values ± SD of groups compared by analysis of variance.

** p value<0.001 of Clinical pregnancy group compared with preclinical abortion.

°° p value <0.001 of Clinical pregnancy group compared with not pregnant group.

Estimation of E2 levels have shown that higher levels are found to be associated with better ovarian response.^18^ The number of oocytes recovered, fertilized and the number of embryos available for cryopreservation were directly proportional to the E2 levels in a study and number of oocytes available for cryo preservation.^5^^,^^17^ Cycles with highest rise of peak E2 were associated with higher number of oocytes and fewer ampoules of puregon which is comparable to results of our study.^19^

The role of high peak E2 levels with improvement in FR has been controversial .In a study, number of oocytes retrieved, number of mature oocytes and FR were not affected by a decline in post E2 levels.^5^ Our study however resvealed that patients who had high peak E2 showed better FR.

In the process of implantation a series of developmental phases occur starting from blastocysts hatching, apposition, adhesion and attachment to the endometriumoccur.^20^ It has been found that during COS, increased E2 produced by growing follicles increases endometrial receptivity, hence chances of implantation required for a successful outcome.^21^ The results of our study highlighted the importance of E2 with a high IR which is supported by Kondapalli et al. in which high E2 levels were associated with better IR, PR and live birth rates.^5^

High responders have been identified by infertility specialists as females with high peak E2 and better oocyte recovery and embryo quality.^22^ In our study, females who had greater number of oocytes produced greater E2 which helped in implantation and attainment of pregnancy. Rehman et al observed that obese females who had a low peak E2 (obese) had poor follicular response in terms of oocyte recovery, embryo quality and endometrial thickness.^4^ The best results in terms of potential of pregnancy was acquired at E2 levels >4000 pg/ml by a group of workers whereas in our study a level of 2269 pg/ml was associated with pregnancy.^18^ The optimal range of peak E2 level was dependent of age of females with 3000–4000 pg/mL in women with age less than 38 years and 2000–3000 pg/mL for women older than 38 years.^11^ Chenette and workers observed that although maximum PR was acquired at 2,777 pg/mL, yet females with E2 levels of less than or equal to 5,000 pg/mL could be proceeded till OPU and ET.^17^ The contradictory results of a decrease in IR was observed in high responders with a high peak E2 by few researchers.^23^ The decrease in IR of high responders was attributed to decrease in endometrial receptivity of high responders by other researchers.^24^ The role of peak E2 levels on the outcome of IVF-ICSI although has remained controversial and subject of intense debate.^9^^,^^22^^-^^24^ Our study has proved an association of higher peak E2 with better reproductive outcomes supported by many researchers.^4^^,^^17^^,^^18^

The study however is limited, as it has not compared changes in peak E2 with respect to age, type of responders (poor / good) and body mass index (BMI).The need of comparison of the results with mid luteal E2 and assessment of progesterone levels is thus required for reliable prediction of successful pregnancy outcomes so as to counsel patients and prepare them about likelihood of cycle outcome.

## CONCLUSION

Peak E2 levels were comparably high in pregnant females as compared to preclinical abortions and non pregnant group of patients. It was found that patients who had higher peak E2 had better oocyte quality parameters with greater number of retrieved, mature and fertilized oocytes and increased endometrial receptivity demonstrated by thick endometrial thickness. These patients had high FR, IR and PR which suggests a positive association of raised peak E2 with pregnancy outcome after ICSI.

## Authors Contribution:

**Rehana Rehman: **Principal investigator, conceived, design and wrote manuscript.

**Shireen Jawed: **Collected data and wrote manuscript.

**Hina Gul: **Did Statistical analysis and formulated tables.

**Rakhshaan Khan: **Wrote, reviewed and approved the manuscript.
